# Supplementary material for: Molecular evolution of the ependymin-related gene epdl2 in African weakly electric fish
Source: G3 (Bethesda). 2022 Dec 19;13(3):jkac331. doi: 10.1093/g3journal/jkac331 (PMC9997568; doi:10.1093/g3journal/jkac331)
Supplement: jkac331_Supplementary_Data [file jkac331_supplementary_data.zip › Suppl_Tables_&_Figs_G3-2022-403804.pdf]

Tables

Table S1. Details of the sequences used in the EPDR phylogenetic analysis

Species	Gene	Code			Source	Notes
		gene	transcript	protein		
Danio rerio	*epd1*	ENSDARG00000103498	ENSDART00000171617.2	ENSDARP00000134034.1	Ensembl	
Denticeps clupeoides	*epd1*	ENSDCDG00000020214	ENSDCDT00000036790	ENSDCDP00000027731.1	Ensembl	
Salmo salar	*epd1*	ENSSSAG00000073991	ENSSSAT00000133331.1	ENSSSAP00000098870.1	Ensembl	
Takifugu rubripes	*epd1*	ENSTRUG00000014952	ENSTRUT00000038357.3	ENSTRUP00000038220.2	Ensembl	
Cyprinus carpio	*epd2*	ENSCCRG00000012904	ENSCCRT00000025647.1	ENSCCRP00000023616.1	Ensembl	
Pygocentrus nattereri	*epd2*	LOC108432735	XM_017706796.2	XP_017562285.1	NCBI	Splicing disagreement with Ensembl ENSPNAG00000025130
Takifugu rubripes	*epd2*	ENSTRUG00000008083	ENSTRUT00000020238.3	ENSTRUP00000020154.3	Ensembl	Manually curated due to missing exons[b]
Amia calva	*epdl*	AMCG00015468	AMCT00015468	AMCP00015468	NCBI GCA_017591485.1, https://github.com/AndrewWT/AmiaGenomics	Manually curated due to missing exons[b]
Amia calva	*epdl*	AMCG00015463	AMCT00015463	AMCP00015463		Manually curated due to missing exons[b]
Amia calva	*epdl*	AMCG00015472	AMCT00015472	AMCP00015472		
Latimeria chalumnae	*epdl*	ENSLACG00000018325.2	ENSLACT00000025567.1	ENSLACP00000023190.1	Ensembl	
Protopterus annectens	*epdl*	LOC122805608	XM_044075849.1	XP_043931784.1	NCBI	
Rhincodon typus	*epdl*	LOC109918485	XM_020518419.1	XP_020374008.1	NCBI	
Rhincodon typus	*epdl*	LOC109936335	XM_020534949.1	XP_020390538.1	NCBI	Manually curated due to missing exons[b]
Clupea harengus	*epdl1*[a]	ENSCHAG00000013904	ENSCHAT00000031403.1	ENSCHAP00000028894.1	Ensembl	Annotated as 3 genes in NCBI and 1 gene with 7 transcripts in Ensembl. We suspect there

						are two genes in tandem
Danio rerio	*epdl1*	ENSDARG00000076386	ENSDART00000111531.4	ENSDARP00000101021.2	Ensembl	
Denticeps clupeoides	*epdl1*	LOC114788914	XM_028977867.1	XP_028833700	NCBI	Splicing disagreement with Ensembl ENSDCDG00000018927
Paramormyrops kingsleyae	*epdl1*	ENSPKIG00000021470	ENSPKIT00000028285.1	ENSPKIP00000004303.1	Ensembl	
Salmo salar	*epdl1*	ENSSSAG00000002722	ENSSSAT00000005774.1	ENSSSAP00000005381.1	Ensembl	
Scleropages formosus	*epdl1*	ENSSFOG00015021928	ENSSFOT00015034799.1	ENSSFOP00015034421	Ensembl	This gene was retired from Ensembl with no successors. However, blast search and synteny inspection suggest this is *epdl1*
Takifugu rubripes	*epdl1*	ENSTRUG00000012059	ENSTRUT00000030623.3	ENSTRUP00000030506.2	Ensembl	
Danio rerio	*epdl2*	ENSDARG00000055539.7	ENSDART00000077910.7	ENSDARP00000072376.5	Ensembl	
Denticeps clupeoides	*epdl2*	ENSDCDG00000027589	ENSDCDT00000054119	ENSDCDP00000043043.1	Ensembl	
Paramormyrops kingsleyae	*epdl2*	*epdl2.1*	*epdl2.1*	*epdl2.1*	This work. GenBank accession number ON863825	Sanger-sequenced and manually curated. There were sequencing errors in coding homopolymers
Protosalanx hyalocranius	*epdl2*	LS_GLEAN_10003579	LS_GLEAN_10003579	LS_GLEAN_10003579	http://gigadb.org/dataset/100262	Manually curated due to missing exons[b]
Salmo salar	*epdl2*	ENSSSAG00000010021	ENSSSAT00000022032.1	ENSSSAP00000020584.1	Ensembl	
Scleropages formosus	*epdl2*	ENSSFOG00015021850	ENSSFOT00015034652.2	ENSSFOP00015034274.1	Ensembl	
Brienomyrus brachyistius	*epdl3*	LOC125720270	XM_048995478.1	XP_048851435.1	NCBI	Manually curated due to missing exons[b]
Paramormyrops kingsleyae	*epdl3*	ENSPKIG00000018439	ENSPKIT00000023217.1	ENSPKIP00000011278.1	Ensembl	

[a]Classified as a unique *epdl* paralog in the Genomicus gene tree (main text Fig. 2A), but reclassified as *epdl1* by the phylogenetic analysis (main text Fig. 2B)
[b]Sequence is supplied in additional file 1

Table S2. Details of the *epdl2* genes found in every species studied

species	source of epdl2 sequence(s)	*epdl2* genes	gene size (bp)[a]	GenBank accession numbers
Brevimyrus niger	DNA extraction, PCR amplification & multiplexed amplicon sequencing with ONT	*epdl2*	1823	ON863837
Brienomyrus brachyistius	genome assembly available (NCBI Gene ID 125720044)	*epdl2*	2166	-
Campylomormyrus sp.	DNA extraction, PCR amplification & multiplexed amplicon sequencing with ONT	*epdl2*	1801	ON863835
Gnathonemus petersii	DNA extraction, PCR amplification & multiplexed amplicon sequencing with ONT	*epdl2*	1779	ON863834
Gymnarchus niloticus	Unannotated genome assembly available (NCBI bioproject PRJNA423259)	*epdl2*	5280	ON863822
Ivindomyrus marchei	DNA extraction, PCR amplification & multiplexed amplicon sequencing with ONT	discarded[b]	-	-
Marcusenius moori	DNA extraction, PCR amplification & multiplexed amplicon sequencing with ONT	*epdl2*	1808	ON863836
Marcusenius ntemensis	DNA extraction, PCR amplification & multiplexed amplicon sequencing with ONT	*epdl2.1*	1675	ON863842
		epdl2.3	1823	ON863841
		epdl2.4	1806	ON863840
Mormyrops zanclirostris	DNA extraction, PCR amplification & multiplexed amplicon sequencing with ONT	*epdl2*	1869	ON863859
Mormyrus tapirus	DNA extraction, PCR amplification & multiplexed amplicon sequencing with ONT	*epdl2*	1816	ON863833
Paramormyrops curvifrons	DNA extraction, PCR amplification & multiplexed amplicon sequencing with ONT	*epdl2.1*	1676	ON863829
		epdl2.3	1823	ON863831
		epdl2.4	1819	ON863830
Paramormyrops hopkinsi	DNA extraction, PCR amplification & multiplexed amplicon sequencing with ONT	*epdl2.2*	1824	ON863857
		epdl2.3	1793	ON863856
		epdl2.4	1821	ON863858
Paramormyrops kingsleyae (APA)	paralog-specific PCR amplification & Sanger-sequencing	*epdl2.1*	1675	ON863825
		epdl2.2	1814	ON863824
		epdl2.3	1809	ON863823
Paramormyrops kingsleyae (BAM)	DNA extraction, PCR amplification & multiplexed amplicon sequencing with ONT	*epdl2.1*	1675	ON863849
		epdl2.2	1813	ON863847

		epdl2.3	1810	ON863848
Paramormyrops sp. MAG (Type I)	DNA extraction, PCR amplification & multiplexed amplicon sequencing with ONT	*epdl2.2*	1823	ON863850
		epdl2.3	1825	ON863851
		epdl2.4	1815	ON863852
Paramormyrops sp. MAG (Type II)	DNA extraction, PCR amplification & multiplexed amplicon sequencing with ONT	*epdl2.2*	1825	ON863826
		epdl2.4	1817	ON863827
Paramormyrops sp. NGO	DNA extraction, PCR amplification & multiplexed amplicon sequencing with ONT	*epdl2.1*	1676	ON863846
		epdl2.3	1815	ON863845
Paramormyrops sp. OFF	DNA extraction, PCR amplification & multiplexed amplicon sequencing with ONT	*epdl2.3*	1823	ON863844
Paramormyrops sp. SN2	DNA extraction, PCR amplification & multiplexed amplicon sequencing with ONT	discarded[c]	-	-
Paramormyrops sp. SN3	DNA extraction, PCR amplification & multiplexed amplicon sequencing with ONT	*epdl2.3*	1824	ON863843
Paramormyrops sp. SN9	DNA extraction, PCR amplification & multiplexed amplicon sequencing with ONT	discarded[c]	-	-
Paramormyrops sp. SZA	DNA extraction, PCR amplification & multiplexed amplicon sequencing with ONT	*epdl2*	1820	ON863828
Paramormyrops sp. TEN	DNA extraction, PCR amplification & multiplexed amplicon sequencing with ONT	*epdl2.2*	1819	ON863854
		epdl2.3	1805	ON863853
		epdl2.4	1817	ON863855
Paramormyrops sp. TEU	DNA extraction, PCR amplification & multiplexed amplicon sequencing with ONT	discarded[c]	-	-
Petrocephalus simus	DNA extraction, PCR amplification & multiplexed amplicon sequencing with ONT	*epdl2*	1792	ON863832
Pollimyrus adspersus	DNA extraction, PCR amplification & multiplexed amplicon sequencing with ONT	*epdl2*	1551	ON863838
Scleropages formosus	genome assembly available (Ensembl gene ENSSFOG00015021850)	*epdl2*	2918	-
Stomatorhinus ivindoensis	DNA extraction, PCR amplification & multiplexed amplicon sequencing with ONT	*epdl2*	1808	ON863839

[a]From start to stop codon. bp = base pairs
[b]This sample was likely an incorrectly identified *P. kingsleyae*
[c]No *epdl2* genes were successfully amplified and sequenced

Table S3. Additional information on the live specimens used, including the NCBI SRA identifiers of the sequencing reads obtained.
Note: this table is released as a standalone .xlsx file.

Table S4. Primers used to amplify and sequence *epdl2* genes across Mormyridae

primer	sequence (5'-3')	target gene	target clade	PCR annealing temperature (^{o}C)
epdl2.1_1F	CAGCCAGTGCGTCTACCATTTGC	*epdl2.1*	*P. kingsleyae*	71
epdl2.1_1R	AGGAATGAAACGAACAAAAGTTCAGGCAAGT			
epdl2.2_2F	GGTGAACTGCAGGTCTAGTTTG	*epdl2.2*	*P. kingsleyae*	65
epdl2.2_2R	ACAGAAGTTCAGGCAACTTTAACTTC			
epdl2.3_2F	AACCTACAAGGGACTTTGCTAACCC	*epdl2.3*	*P. kingsleyae*	64
epdl2.3_1R	GCCATGGACTACTTCTACAGCGCAG			
epdl2_Sanger_1	TTCCTATCTGCCCTGGTA	*epdl2.1, epdl2.2, epdl2.3*	*P. kingsleyae*	NA
epdl2_Sanger_2	TATCGCTGGGATTTCGAG	*epdl2.1, epdl2.2, epdl2.3*	*P. kingsleyae*	NA
epdl2_Sanger_3	CTGTTATCCTAGGGATGAGG	*epdl2.1, epdl2.2, epdl2.3*	*P. kingsleyae*	NA
epdl2_Sanger_4	CTGTCCAGGTTCTAATGC	*epdl2.1, epdl2.2, epdl2.3*	*P. kingsleyae*	NA
epdl2_Sanger_5	TTCCAGACAAGCTCACTG	*epdl2.1, epdl2.2*	*P. kingsleyae*	NA
epdl2_0408_F01	AGCARCRACACATTTTTGK	all *epdl2* genes	*Ivindomyrus, Marcusenius ntemensis, Mormyrus, Paramormyrops*	60[a]
epdl2_0408_R01	AGGGTTTSGAGTCAGGR			
epdl2_Pol+Sto_F01	GTTGTTTTCAAAGTCGTCC	all *epdl2* genes	*Pollimyrus, Stomatorhinus*	60
epdl2_Pol+Sto_R01	TGAACCTTGGATTCACAC			
epdl2_Bre+Hyp_F01	TTACTTTCAGTGCTGTATC	all *epdl2* genes	*Brevimyrus*	53
epdl2_Bre+Hyp_R01	CAGAGAATGCAGATAATTCAC			
epdl2_Mar+Cam_F01	GTTGTTTTCAAAGTCGTCC	all *epdl2* genes	*Campylomormyrus, Gnathonemus, Marcusenius*	52, 53, 55[b]
epdl2_Mar+Cam_R01	TCTCTCTCCCTCTGATATA			
epdl2_Morps_F01	CGAATCCTTAAATCCCAATC	all *epdl2* genes	*Mormyrops*	55
epdl2_Morps_R0	CTGTAACGATTCACATGA			

1	C			
epdl2_Pet_F01	AGGGACAAYTTAGTCAG GA	all *epdl2* genes	*Petrocephalus*	60
epdl2_Pet_R01	TTGAGTCAGAGRACACA GT			

NA: primer not used in PCR reactions
[a]Touch-up annealing temperatures for: *Paramormyrops curvifrons*, *Paramormyrops* sp. OFF, *Paramormyrops* sp. SN2, *Paramormyrops* sp. SN9, *Paramormyrops* sp. TEU. Touch-up conditions: one set was comprised of 7 cycles from 57 to 60°C in 0.5°C increments. Each PCR consisted of 4 sets followed by 4 additional cycles at 60°C.
[b]*Campylomormyrus*: 52°C, *Gnathonemus*: 55°C, *Marcusenius*: 53 °C

Table S5. PCR reagents and final concentrations used to amplify each *epdl2* gene in *P. kingsleyae*

Reagent	Final concentration
5x Q5 reaction buffer	1x
dNTPs	200 µM each dNTP
Forward and Reverse Primers	1.0 µM each
Template DNA	<1000 ng
Q5 polymerase	0.02 U/µl
5x GC enhancer for Q5	0.5x

Table S6. PCR conditions used to amplify each *epdl2* gene in *P. kingsleyae*

Step	temperature (°C)	time
Preheat lid + block	98	-
Initial denaturation	98	30 s
30 cycles of:		
denaturation	98	10 s
annealing	primer-dependent (Table S4)	25 s
extension	72	2 min
Final extension	72	2 min

Table S7. Mormyrid species and their NBCI bioproject codes that guided *epdl2* primer design

species	BioProject
Boulengeromyrus knoepffleri	PRJNA526756
Brevimyrus niger	PRJNA526749
Genyomyrus donnyi	PRJNA529465
Gnathonemus echidnorhynchus	PRJNA529468
Hyperopisus bebe	PRJNA529477
Isicthys henryi	PRJNA529470
Ivindomyrus marchei	PRJNA529476
Marcusenius schilthuisiae	PRJNA529469
Mormyrops attenuatus	PRJNA530793
Mormyrops boulengeri	PRJNA530782
Mormyrops zanclirostris	PRJNA530797
Mormyrus hasselquistii	PRJNA542939
Mormyrus iriodes	PRJNA542943
Mormyrus proboscirostris	PRJNA530791
Myomyrus macrops	PRJNA423275
Myomyrus pharao	PRJNA547756
Paramormyrops hopkinsi	PRJNA547741
Paramormyrops sp. MAG	PRJNA547743
Petrocephalus microphthalmus	PRJNA423286
Petrocephalus schoutedeni	PRJNA547742
Petrocephalus sullivani	PRJNA427158
Petrocephalus zakoni	PRJNA547751
Pollimyrus isidori	PRJNA547785
Pollimyrus plagiostoma	PRJNA547754
Stomatorhinus walkeri	PRJNA547748

Table S8. PCR reagents and final concentrations used to amplify all *epdl2* genes across Mormyridae

Reagent	Final concentration
5x Q5 reaction buffer	1x
dNTPs	200 µM each dNTP
Forward and Reverse Primers	0.9 µM each[a] or 0.5 µM each[b]
Template DNA	<1000 ng
Q5 polymerase	0.02 U/µl
5x GC enhancer for Q5	0.1x

[a]epdl2_0408 primers with *Ivindomyrus marchei*, *Marcusenius ntemensis*, and all *Paramormyrops* spp
[b]epdl2_0408 primers with *Mormyrus tapirus*, and all other primers

Table S9. PCR conditions used to amplify all *epdl2* genes across Mormyridae

Step	temperature (^{o}C)	time
Preheat lid + block	98	-
Initial denaturation	98	30 s
30[a] cycles of:		
denaturation	98	10 s
annealing	primer- and species-dependent (Table S4)	20 s[b]
extension	72	75 s[c]
Final extension	72	2 min

[a]32 cycles for: *Brevimyrus niger*, *Campylomormyrus* sp, *Ivindomyrus marchei*, *Marcusenius ntemensis*, *Paramormyrops curvifrons*, *Paramormyrops* sp. OFF, *Paramormyrops* sp. SN2, *Paramormyrops* sp. SN9, *Paramormyrops* sp. TEU
[b]25 seconds for: *Brevimyrus niger, Campylomormyrus* sp, *Ivindomyrus marchei*, *Marcusenius ntemensis*
[c]90 seconds for: *Brevimyrus niger, Campylomormyrus* sp

Table S10. Sites along Epdl2 that have experienced positive selection in the osteoglossiform *epdl2* gene tree and have evolved at increased ω rates in mormyrid lineages with vs without *epdl2* duplications. Branches supported by each EBF value are marked by rectangles in Fig. S3 (EBF values for a given site are arranged left to right to match their corresponding branches from top to bottom)

Site	Estimated number of branches under positive selection	Empirical Bayes Factor (EBF)
28	1	1.1×10^{4}
105	1	2.6×10^{11}
106	0	-
125	1	5.5×10^{4}
126	2	1449, 1.0×10^{26}
127	2	2200, 3.2×10^{10}
129	2	543, 1.0×10^{26}
150	2	2165, 315
153	1	8.8×10^{12}
172	4	2.4×10^{4}, 2.4×10^{4}, 1.0×10^{26}, 1043

Table S11. Summary of the amino acid substitutions observed in the Epdl2 paralogs at the ten sites under positive selection and increased ω rates in mormyrid lineages with vs without *epdl2* duplications

site	ancestral amino acid	derived amino acid residues observed in each paralog (total)			
		epdl2.1 (5)	*epdl2.4* (6)	*epdl2.2* (6)	*epdl2.3* (10)
28	S	-	-	P (4)	P (10)
105	F	-	-	S (1)	-
106	P	R (5)	L (6)	R (6)	R (10)
125	S	-	N (1)	N (6)	N (10)
126	S	-	-	-	L (8)
127	A	-	-	D (6)	-
129	G	-	S (1)	S (2)	S (2)
150	Q	L (5)	L (4), K (2)	K (6)	K (10)
153	F	-	-	C (1)	-
172	L	-	-	R (1)	W (7), R (2)

Figures

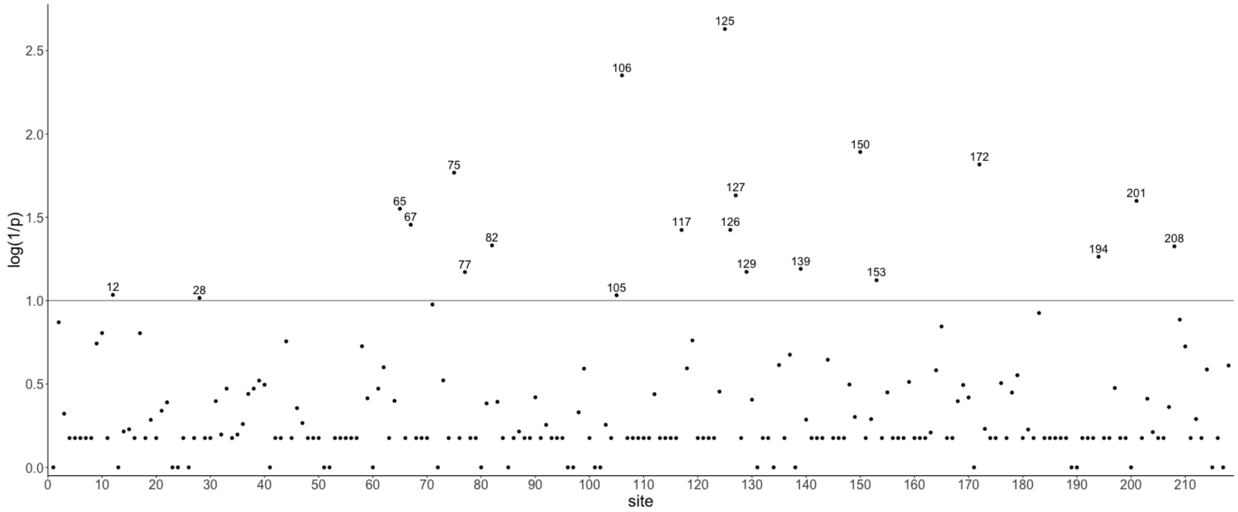

Fig. S1. Sites along Epdl2 that have experienced positive selection (MEME, p < 0.1) in the osteoglossiform taxa studied. p values have been transformed so that higher values on the y axis represent lower p values. Horizontal line marks the significance threshold and significant sites are labeled.

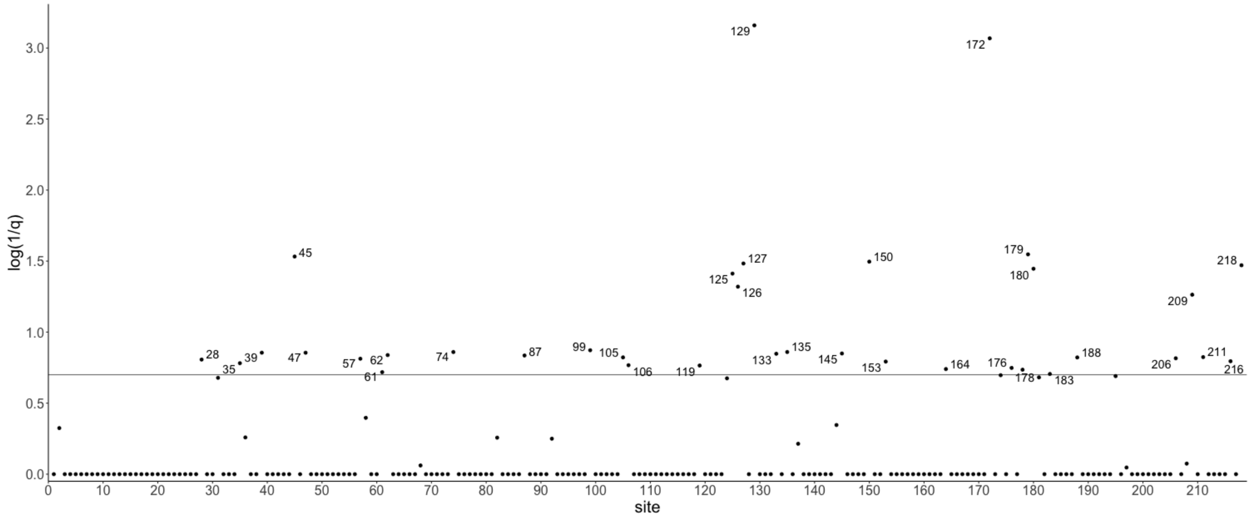

Fig. S2. Sites along Epdl2 with higher ω values in mormyrid lineages with vs without epdl2 duplications (Contrast-FEL, q < 0.2). q values have been transformed so that higher values on the y axis represent lower q values. Horizontal line marks the significance threshold and significant sites are labeled.

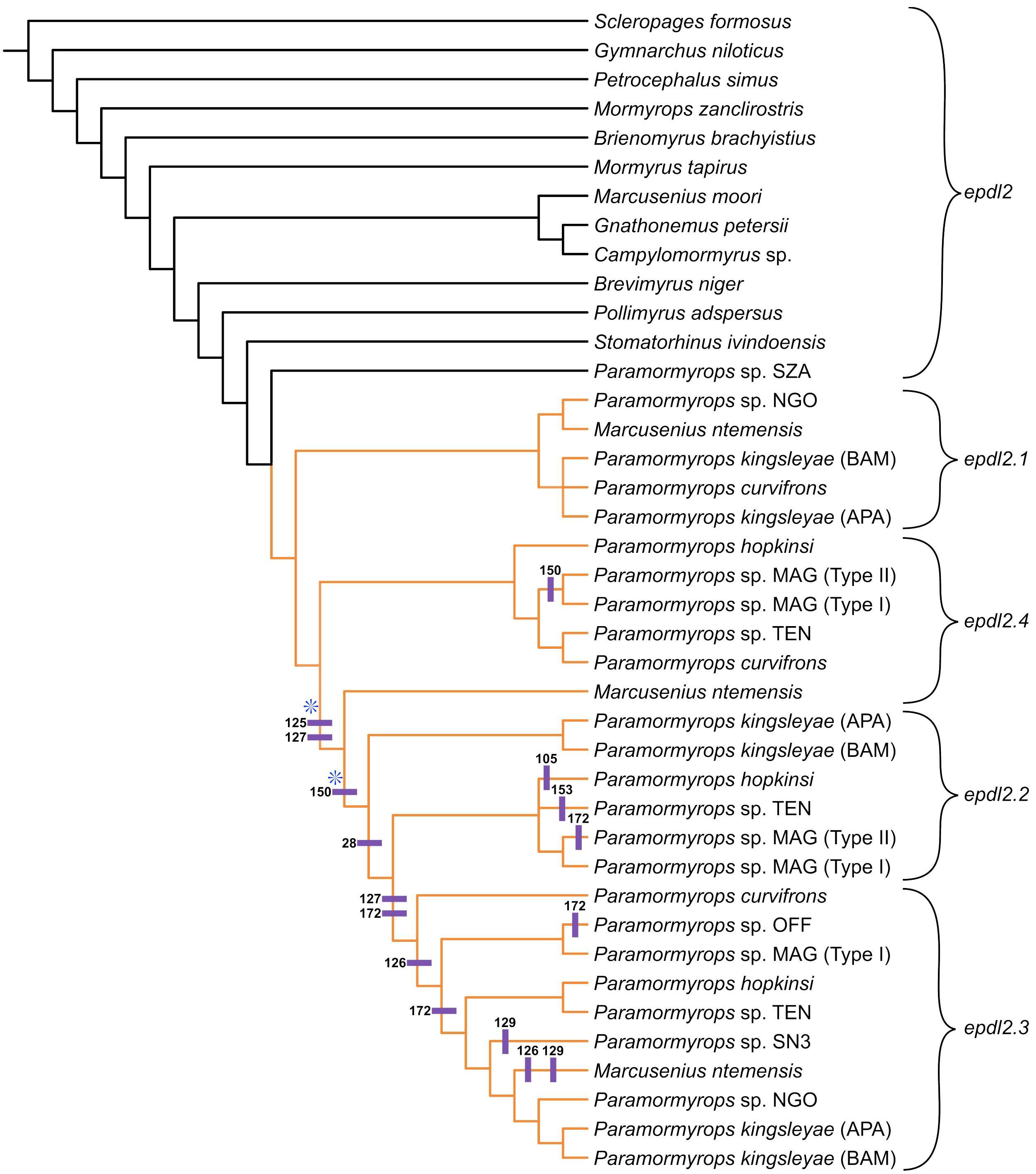

Fig. S3. Topology of the osteoglossiform *epdl2* gene tree, we highlight branches and sites where we detected signals of selection. Orange branches are recently duplicated *epdl2* paralogs, selection has intensified in these branches relative to the mormyrid lineages without *epdl2* duplications. Branches with blue asterisks experienced positive selection. Purple rectangles are labeled with sites along Epdl2 where selection has intensified in mormyrid lineages with *epdl2* duplications and have experienced positive selection. These rectangles are placed on the branches where exploratory evidence suggests they underwent positive selection. All rectangles map to the lineages with *epdl2* duplications.
